# Hospital-Acquired Pressure Injury Reduction: A Nurse-Led Quality Improvement Initiative in Qatar

**DOI:** 10.7759/cureus.81726

**Published:** 2025-04-04

**Authors:** Thabit Melhem, Valarmathi Varadharajan, Ian S Mcdonald, Mariam N Al-Mutawa, Dyna George, Dona Thomas, Nabila Chaabna, Abdulqadir J Nashwan

**Affiliations:** 1 Nursing and Midwifery Department, Hamad Medical Corporation, Doha, QAT; 2 Nursing Department, Heart Hospital, Hamad Medical Corporation, Doha, QAT; 3 Nursing and Midwifery Research Department, Hamad Medical Corporation, Doha, QAT

**Keywords:** comprehensive unit-based safety program, hospital-acquired pressure injuries, nurse-led, qatar, quality improvement

## Abstract

Hospital-acquired pressure injuries (HAPIs) pose a considerable challenge for healthcare systems, not only for their impact on patient well-being but also for the significant financial burden imposed on healthcare facilities. Between 2020 and 2021, Hamad Medical Corporation (HMC) reported an average of 60±5 monthly HAPI incidents, with medical devices contributing to approximately 50% of these cases. This project aimed to reduce the incidence of pressure injuries across hospital units. A quality improvement (QI) project using the Comprehensive Unit-Based Safety Program (CUSP) model was implemented to reduce HAPI incidence by 50% by September 2022. The interventions included staff training, electronic documentation review, spot audits on SSKIN (surface, skin inspection, keep moving, incontinence, and nutrition) care bundle compliance policy updates, and enhanced utilization of pressure-relieving devices. The project resulted in a 64.4% reduction in HAPI incidence, with a 66% decrease specifically in medical device-related pressure injuries. The nurse-led, evidence-based interventions significantly reduced HAPI rates across HMC facilities, demonstrating the effectiveness of targeted quality improvement efforts and offering a model that other healthcare institutions can adapt.

## Introduction

Hospital-acquired pressure injuries (HAPIs) are localized damage to the skin and underlying tissue that occurs during a hospital stay. These preventable injuries are caused by the prolonged compression over patients’ bony structures and are classified into six stages according to severity [1,2]. HAPIs substantially burden healthcare systems due to their implications for patient care, healthcare costs, and clinical practices [3,4]. The Agency for Healthcare Research and Quality (AHRQ) estimates that treating hospital-acquired pressure injuries costs between $20,900 and $151,700 per patient [5]. In addition, HAPIs significantly extend patients’ hospital stays by 23.9 days [6]. The development of these injuries can also lead to serious complications, including readmission, infection, and death [4-7].

Globally, the incidence and prevalence of HAPIs remain a significant concern. A systematic review and meta-analysis by Li et al. estimated the pooled HAPI rate of 8.4% (95% CI 7.6-9.3%) [8]. In Qatar, the incidence of pressure injury was estimated to be 6.1/per 1000 patient days [9], which reflects the global challenge. The measures of HAPI cases are captured by the National Database of Nursing Quality Indicators (NDNQI) and are classified as nursing-sensitive indicators [10]. These measures are tracked and benchmarked against national standards for continuous improvement in nursing practices.

Nurses play a central and irreplaceable role in preventing, assessing, and managing pressure injuries, owing to their continuous and direct contact with patients. Their clinical judgment, vigilance in risk identification, and timely interventions form the foundation of effective pressure injury prevention strategies. Recent evidence underscores that nurses' knowledge, clinical competence, and systematic skin assessment skills are significantly associated with reduced incidence rates of HAPIs [11,12]. For instance, nurses who demonstrate proficiency in early risk identification and consistently apply evidence-based practices (such as repositioning, use of support surfaces, and nutritional assessment) are more effective in mitigating injury development. However, despite widespread acknowledgment of their critical role, persistent gaps in knowledge, variable adherence to clinical guidelines, and inconsistency in implementing preventive measures have been reported across healthcare settings. These deficiencies may stem from limited access to structured training, high staff turnover, and organizational culture and leadership support. As a result, ongoing professional development, competency-based training, and structured quality improvement (QI) initiatives remain essential to empower nurses, enhance consistency in care delivery, and ultimately reduce the burden of pressure injuries.

This article was previously presented as a meeting abstract at the Qatar Health Congress 2023 and the 3rd Qatar Public Health Conference on November 25, 2023.

## Materials and methods

Root cause analysis

**Figure 1:**
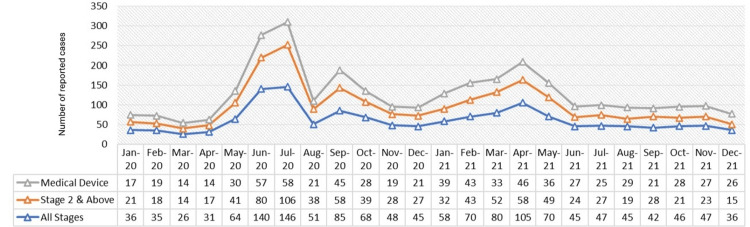
HAPI incidences before intervention. HAPI: Hospital-acquired pressure injury. Image credit: Thabit Melhem.

HAPIs are preventable; however, their high occurrence requires appropriate interventions to mitigate their impact on patient outcomes and healthcare resources. At Hamad Medical Corporation (HMC), the largest healthcare provider in Qatar, the incidence of HAPIs of all stages from 2020 to 2021 was estimated to be around 60±5 cases per month. Medical devices contributed to almost 50% of this estimate, with devices such as nasal masks, bilevel positive airway pressure (BIPAP), nasal cannulas, Hollister ties, and nasogastric tubes being the top five devices contributing to HAPIs. Figure 1 illustrates the overall distribution of HAPI cases, including all stages, stage II and above, and medical device-related HAPI incidences.

**Figure 2:**
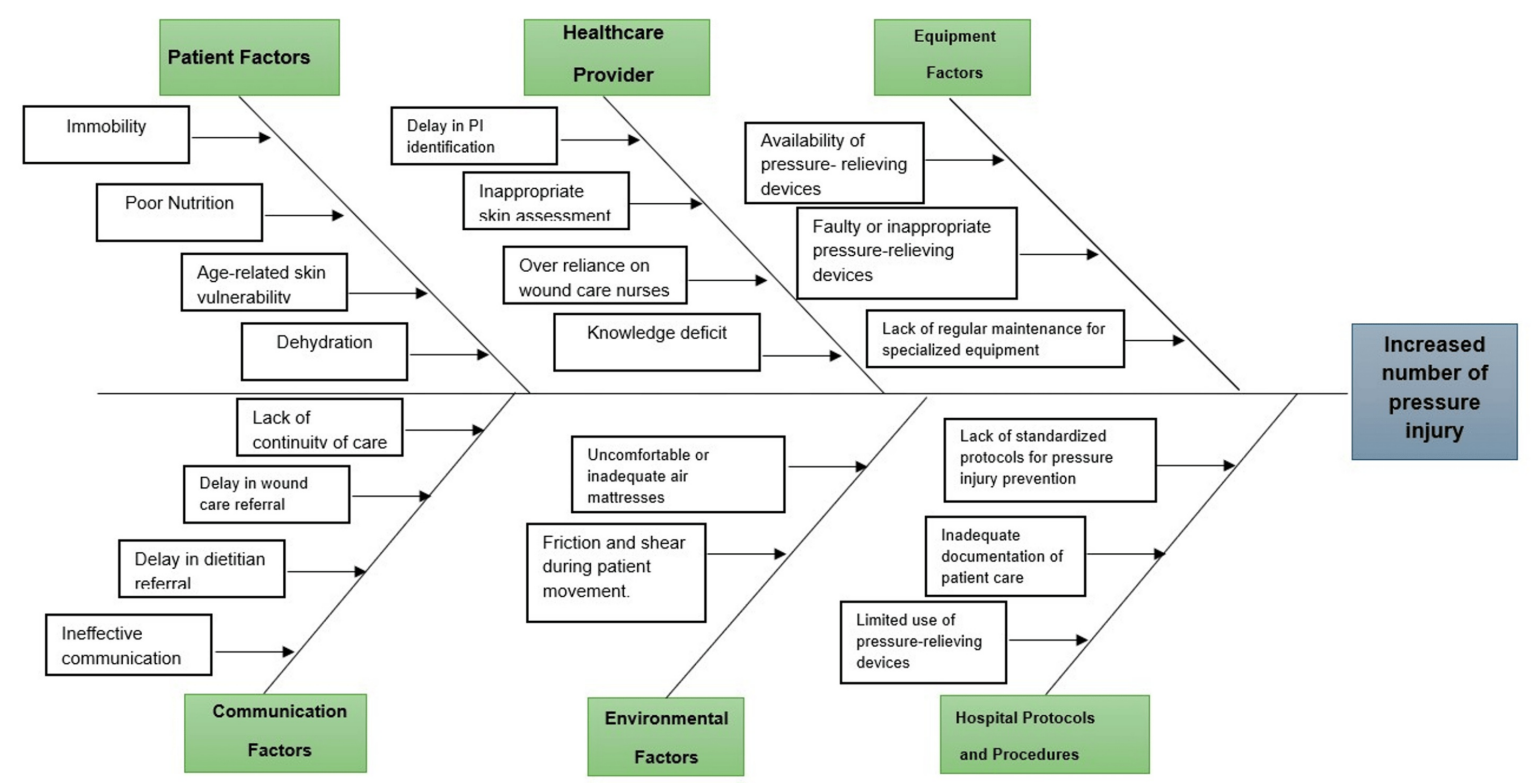
Fishbone (cause and effect) diagram. Image credit: Thabit Melhem.

A root cause analysis conducted by the Corporate Nursing and Midwifery team and selected facility nursing leadership at HMC identified key factors contributing to pressure injuries (Figure 2). These factors included frontline knowledge gaps on pressure injury identification, staging, prevention, and management; disparities in nursing practices related to prevention strategies; inconsistent and ineffective use of pressure injury-relieving devices; and front-line staff's overreliance on wound care nurses. Additionally, there was a lack of continuity in the care of patients with pressure injuries during transfers between the Emergency Department, inpatient areas, and the community. Other contributing factors included delays in reporting and escalating HAPI incidences to unit leadership and relevant stakeholders, late or delayed referrals to wound care, and challenges in patient repositioning due to clinical conditions.

Intervention design

Based on the findings from this comprehensive analysis, a patient-centered, evidence-based quality improvement initiative at HMC was launched in two phases across 12 HMC facilities starting in December 2021. This quality improvement project aimed to reduce HAPI and medical device-related pressure injury (MDRPI) incidence rates by 50% across HMC by September 2022.

Data collection

Data collection for this project was performed monthly using audits, electronic medical records, the e-incident reporting system, and the Nursing and Midwifery Data Management System (NMDMS). All data collection was conducted at HMC. The HAPI incidence rate was the primary outcome measure (Table 1).

**Table 1:** Project measures. RL: Risk and learning; MS: Microsoft; SSKIN: S – Surface, S – Skin inspection, K – Keep moving, I – Incontinence/moisture, N – Nutrition and hydration; HAPI: hospital-acquired pressure injury; MDPRI: Medical device-related pressure injury. ^a^ [13], ^b^ Internal document, Hamad Medical Corporation – Nursing and Midwifery Department, Quality Improvement Unit (unpublished).

Percentage compliance with SSKIN care bundle^a^	Process measure	The number of patients assessed for all SSKIN care bundle^a^ preventive measures using the Spot audit checklist	The number of patients in the unit at risk of developing pressure injuries was assessed using the Spot audit checklist^b^ for SSKIN bundle^a^ preventive measures	Collected from MS Forms (Microsoft Corp., Redmond, WA) Spot audit collection tool^b^
Percentage of staff who completed the HAPI educational module	Process measure	No. of staff who completed the HAPI educational module	No. of staff nominated for the session	Nursing and Midwifery Education Department - educational attendance records
Pressure ulcer incidence	Outcome measure	Total number of pressure ulcer incidence reported through RL Solutions (Toronto, Canada)	Total number of patient days for the month	Collected from RL Solutions (Toronto, Canada) by facility quality lead
Pressure ulcer incidence	Outcome measure	Total number of pressure ulcer incidences - stage 2 and above - were reported through RL Solutions (Toronto, Canada)	Total number of patient days for the month	Collected from RL Solutions (Toronto, Canada) by facility quality lead
Pressure ulcer incidence	Outcome measure	Total number of pressure ulcer incidence - MDRPI reported through RL Solutions (Toronto, Canada)	Total number of patient days for the month	Collected from RL Solutions (Toronto, Canada) by facility quality lead

Implementation strategy

To monitor the process, we have selected the percentage of staff who completed the HAPI educational module in the defined period and the percentage of compliance with the SSKIN (surface, skin inspection, keep moving, incontinence, and nutrition) care bundle [13]. To measure compliance, we have selected the patients at risk for developing pressure injuries using risk assessment tools such as the Braden scale for adults [14], the Braden Q scale for pediatrics [15], and the Neonatal Tissue Viability Risk Assessment scale [16]** **for neonates. Baseline data showed an incidence rate of 0.89 HAPI cases per 1,000 patient days for all stages, with specific monitoring of Stage 2 and higher and medical device-related pressure injuries.

The Corporate Nursing and Midwifery Department collaborated with multidisciplinary teams to initiate a patient-centered, evidence-informed quality improvement project at HMC. The team involved nurses, doctors, and quality improvement specialists. The Comprehensive Unit-Based Safety Program (CUSP), AHRQ approach was used as a quality improvement model [17], with unit-based HAPI champions (at HMC) leading the initiative at the unit level. The project used the four-step framework (engage, educate, execute, and evaluate) [18].

The CUSP model is a well-established quality improvement model designed to enhance patient safety by improving healthcare workers' safety culture and adherence to evidence-based practices. CUSP focuses on promoting teamwork, identifying safety risks, and implementing solutions. It combines frontline staff engagement with leadership support to address unit-specific safety issues. The model typically follows steps like identifying defects, engaging leadership, and evaluating results to sustain improved safety outcomes, such as infection prevention and reducing hospital-acquired conditions.

The 4E model stands for engage, educate, execute, and evaluate and is often used to drive change in clinical practices, including patient safety efforts [18]. The 4E implementation model and the CUSP model are related in their goals to improve patient safety and healthcare quality [17]. While CUSP identifies and addresses safety issues, the 4E framework provides clarity and organization to the execution strategies through its systematic approach. Both frameworks underscore the importance of sustainability; however, the 4E model’s “Evaluate” phase introduces an additional tier of structured feedback, facilitating ongoing monitoring and modifications to uphold enhancements over time. Together, the 4E and CUSP models enhance one another by promoting staff engagement, education, and ongoing assessment, forming a robust foundation to maintain improvements in patient safety.

Brainstorming sessions helped identify key interventions, focusing on curriculum standardization, staff education, pressure-relieving devices, and patient and family involvement. In addition, a project charter and action plan were created to define the goals, objectives, performance measures, and timelines, providing a clear roadmap for the initiative.

The 4E implementation framework guided the project's implementation. It facilitated education, collaboration, and standardized interventions and ensured that the project’s goals were consistently met across all HMC facilities.

Engage: In September 2020, a multidisciplinary project team was formed to address the rising concern of HAPI. The team included nurses, wound care specialists, dietitians, respiratory therapists, occupational therapists, nursing informatics, and educators. To facilitate communication and enhance project execution, HAPI leads were appointed from each facility, and unit-based HAPI champions were identified to oversee project activities at the unit level. The roles and responsibilities of these champions, facility leads, and respiratory therapy champions were clearly defined. A comprehensive communication plan and reporting governance structure were developed to ensure that all relevant stakeholders were informed about the project’s progress.

Educate: A standardized curriculum was implemented across all facilities to equip the staff with the necessary skills and knowledge. This curriculum incorporated evidence-based best practices for HAPI prevention, identification, staging, and management. The curriculum also integrated the management of medical devices contributing to pressure injuries. A comprehensive clinical simulation-based education program was rolled out, where 226 HAPI champions were trained in two phases from September to November 2021 and March to June 2022. Facility educators provided additional unit-based education sessions, and supplementary sessions were organized to train 44 newly identified champions during the COVID-19 pandemic and FIFA World Cup 2022.

Execute: In the execution phase, existing clinical guidelines for pressure injury prevention and management were revised to integrate best practices for HAPI prevention with a focus on medical device-related pressure injuries. Various monitoring strategies were introduced to evaluate the project’s progress, such as compliance checks for the SSKIN bundle, development of performance measures to monitor HAPI in perioperative areas, monthly tracking of HAPI occurrences by stages, and electronic report-related pressure injury prevention documentation from patients’ electronic medical records. Regular facility visits were conducted to observe unit-level practices, and monthly meetings with facility leads and HAPI champions were held to address any issues and reinforce good practices. A medical device monitoring sheet was also introduced to optimize pressure-relieving devices across all units and facilities.

Evaluate: The HAPI project evaluation incorporated escalation and comprehensive assessment measures to ensure issues were promptly addressed, strategies were effectively implemented, and outcomes were achieved. A structured reporting and escalation system enabled timely communication of audit issues to the nursing leadership. Regular HAPI meetings discussed challenges, successes, and areas for improvement. An incident review process with case review templates identified practice gaps while facility leads collaborated with nursing leadership to secure pressure-relieving devices, supporting sustainable HAPI prevention improvements. A parallel assessment of the project’s strategies and outcomes was conducted. This evaluation utilized process and outcome indicators, such as compliance with the SSKIN bundle, completion of the HAPI education module, and incidence rates of pressure injuries (including stage 2 and above and those associated with medical devices). The project outcomes were analyzed using 'run chart rules' to detect significant changes over time, and a comparative analysis was performed between the pre-intervention and post-intervention phases to assess the impact of the interventions. These combined efforts in escalation and evaluation reinforced the effectiveness and sustainability of the HAPI prevention strategies.

Data analysis

Data were analyzed using descriptive and comparative statistical methods to evaluate the impact of the interventions. Monthly HAPI incidence rates (per 1,000 patient days) were calculated and compared between the pre-intervention (2020-2021) and post-intervention (December 2021 - September 2022) periods. Run chart analysis was used to identify trends and significant changes over time. The comparative analysis calculated the percentage reduction in overall HAPI cases and MDRPI. Statistical significance was determined using paired t-tests and chi-square tests where applicable. Process measures, including compliance with the SSKIN care bundle and completion rates of the HAPI educational module, were analyzed to assess the consistency and effectiveness of implementation. Outcome measures, such as reducing stage II and above pressure injuries and MDRPI rates, were evaluated to determine the project's overall success. All analyses were conducted using IBM SPSS Statistics for Windows, Version 26 (IBM Corp., Armonk, NY).

## Results

During the two implementation phases, Phase 1 (September to November 2021) and Phase 2 (March to June 2022), 85% of the targeted staff members completed the one-hour unit-based educational sessions. This target was met by June 2022, ensuring all staff received standardized education.

Initial compliance with the SSKIN care bundle across all hospitals began at 91.1% and fluctuated over time. The HAPI champions and facility leadership used the compliance reports to identify challenges and enhance compliance levels with the SSKIN care bundle. In addition, electronic medical reports validated SSKIN care bundle compliance.

**Figure 3:**
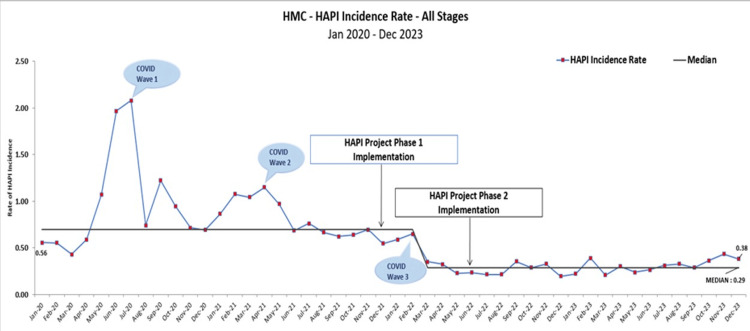
HAPI incidences pre- and post-implementation. HAPI: Hospital-acquired pressure injury; HMC: Hamad Medical Corporation. Image credit: Thabit Melhem.

**Figure 4:**
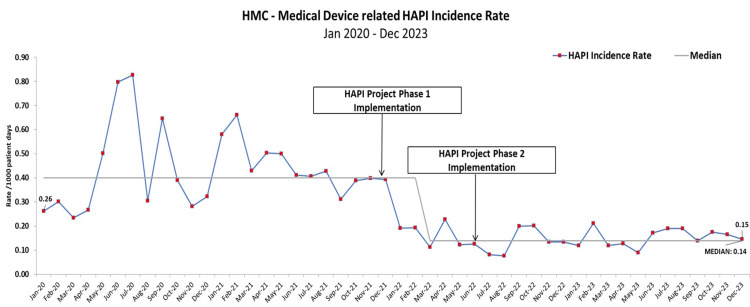
Medical device-related HAPI incidences, pre- and post-implementation. HAPI: Hospital-acquired pressure injury; HMC: Hamad Medical Corporation. Image credit: Thabit Melhem.

**Figure 5:**
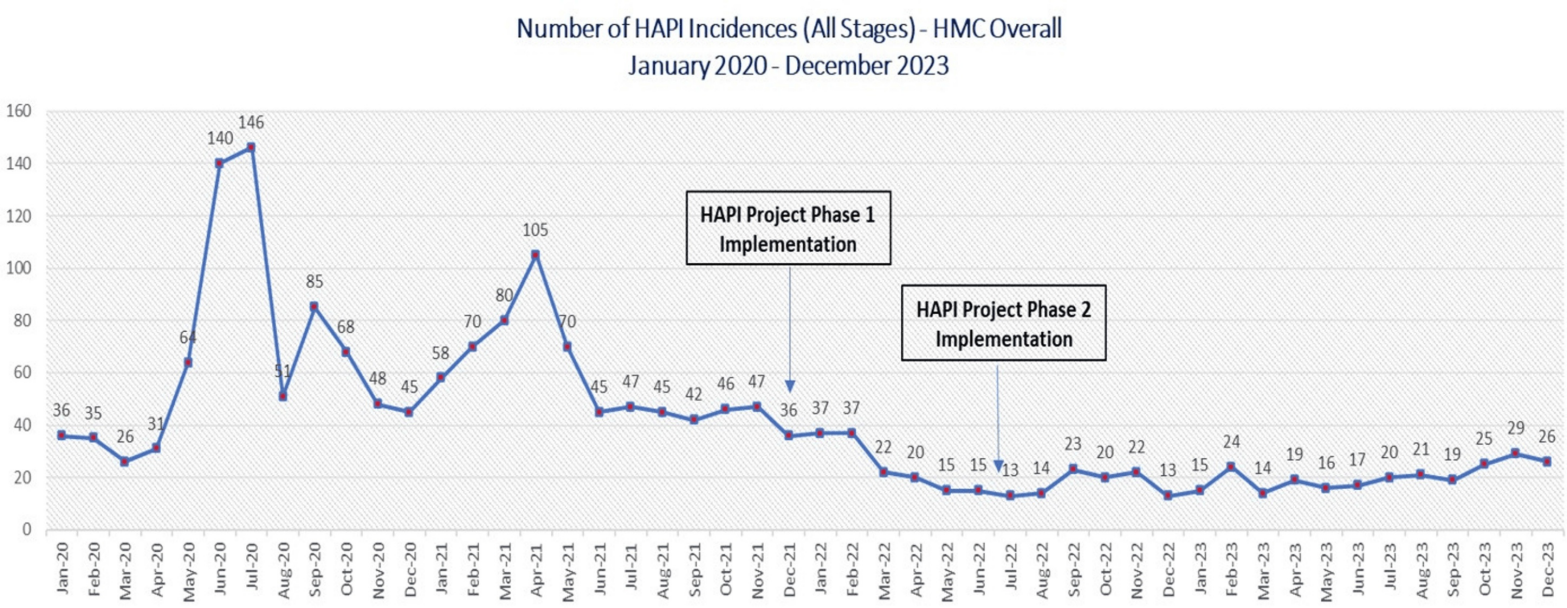
Number of HAPI incidences, pre- and post-implementation. HAPI: Hospital-acquired pressure injury; HMC: Hamad Medical Corporation. Image credit: Thabit Melhem.

The project successfully reduced HAPI incidence rates across the board (Figures 3-5). The incidence rate for all stages of HAPI had fallen by 64% from 0.89 cases per 1,000 patient days reported between January 2020 and December 2021 to 0.32 cases per 1,000 reported from January 2022 to December 2023. The average monthly reported cases dropped from 61 in the pre-intervention period to 21 in the post-intervention period. A similar trend was observed in the more severe cases (Stage II and above), where the rate decreased by 66%. The incidence rate declined from 0.53 cases per 1,000 patient days in the pre-intervention stage to 0.18 cases per 1,000 patient days in the post-intervention stage. The monthly average of these cases dropped from 36 to 12. Pressure injuries caused by medical devices also showed a significant reduction of 65.91%. The incidence rate fell from 0.40 cases per 1,000 patient days in the pre-intervention phase to 0.14 cases per 1,000 patient days in the post-intervention phase. This drop reflected the decrease in the average number of cases from 30 to just 10 during the same period.

## Discussion

This project highlighted the importance of staff engagement and continuous monitoring to ensure the efficacy of HAPI prevention strategies. A key lesson learned was the critical role of frontline nurse engagement and the leadership of HAPI champions in driving project success. Keeping frontline nurses informed through unit quality boards and electronic data systems enhanced their empowerment and accountability, contributing to the project's overall success [5,19]. However, the project faced several limitations.

Logistical challenges were notable, particularly for conducting the COVID-19 pandemic-imposing and facilitating regular meetings. Additional limitations included staffing constraints due to the deployment of HAPI champions and frontline nurses during the COVID-19 pandemic and FIFA World Cup 2022, which affected the availability of personnel for HAPI audits and ongoing education. As bedside nurses providing patient care, HAPI champions faced difficulty dedicating time to reviewing daily reports and conducting spot audits [20].

Measures to ensure the sustainability of the project’s outcomes were implemented, including ongoing training, regular audits, and regular updates to policies and guidelines. Furthermore, HAPI champions would continue to lead the effort in their respective units, ensuring adherence to the SSKIN care bundle and evidence-based pressure injury practices [21-23]. From a practice perspective, this project demonstrates the effectiveness of empowering frontline nurses through structured education, leadership engagement, and data-driven feedback loops. Embedding such nurse-led quality improvement models into routine clinical governance frameworks can drive a culture of accountability, enhance interdisciplinary collaboration, and promote consistent preventive care. Moreover, integrating real-time monitoring tools and unit-level champions ensures that best practices are sustained beyond the intervention period, supporting continuous learning and improvement. This model can be replicated in other healthcare systems seeking to reduce hospital-acquired harm while fostering a proactive, nurse-led patient safety culture.

Despite the project's overall success, several limitations were identified that may have influenced the outcomes. The logistical challenges posed by the COVID-19 pandemic and the FIFA World Cup 2022 significantly impacted the implementation of the project, particularly in conducting simulation-based training sessions and holding regular meetings due to operational pressures and staffing shortages. Many HAPI champions and frontline nurses were redeployed to meet the increased demands during these events, which affected their availability for educational sessions and audit activities. Furthermore, bedside nurses and HAPI champions faced time constraints in reviewing daily reports and conducting spot audits due to their patient care responsibilities. This may have affected the consistency and thoroughness of data collection and intervention monitoring [5]. Variation in compliance with the SSKIN care bundle across different hospital units was another challenge, influenced by unit workload, staff turnover, and differences in leadership engagement. A multi-pronged strategy has been established to support long-term sustainability, including institutionalizing periodic refresher training, reinforcement of audit mechanisms, and integration of pressure injury prevention into routine quality metrics and staff performance evaluations. Facility-based HAPI champions remain central to this approach, promoting ongoing mentorship, peer support, and timely escalation of concerns. Leadership engagement continues to be reinforced through monthly dashboards, regular feedback loops, and facility-level accountability structures. These strategies aim to sustain the initial gains and foster a resilient safety culture capable of adapting to workforce and operational changes.

Data reporting and accuracy were also potential limitations, as reliance on electronic documentation introduced the risk of incomplete data entry or misclassification of pressure injuries [20]. Delays in incident reporting may have also affected the accuracy of real-time data analysis. While the project demonstrated promising results, sustaining these improvements long-term will require ongoing staff education, leadership support, and consistent monitoring of compliance with HAPI prevention protocols [22]. Addressing these limitations in future initiatives will ensure the continued success and sustainability of HAPI prevention efforts across healthcare settings.

## Conclusions

In conclusion, the HAPI reduction project successfully reduced the incidence of HAPIs through effective staff engagement, education, and implementation of evidence-based interventions. Despite challenges related to staffing shortages, the project achieved its objectives and improved patient outcomes. Future efforts should ensure the long-term sustainability of the project’s outcomes. The sustained commitment of HAPI champions and consistent implementation of regular audits will be critical for maintaining these improvements and reducing the burden of HAPIs across the healthcare system.
